# Arterial Wall Compliance of the Lower Limb Arteries in Healthy Adults—A Systematic Review and Meta-Analysis

**DOI:** 10.3390/jcm15176756

**Published:** 2026-08-31

**Authors:** Cathryn Bassett, Bernhard Hruschka, Saurav Ranjan Mohapatra, Jelle Frankort, Pauline Riedl, Eva Kalkum, Christian Uhl, Alexander Gombert, Panagiotis Doukas

**Affiliations:** 1 Department of Vascular Surgery, University Clinic RWTH Aachen, Pauwelsstr. 30, 52074 Aachen, Germany; 2 Institut für Textiltechnik, RWTH Aachen University, Otto-Blumenthal-Straße 1, 52074 Aachen, Germany; 3 Study Center of the German Society of Surgery (SDGC), University of Heidelberg, Im Neuenheimer Feld 130.3, 69120 Heidelberg, Germany

**Keywords:** arterial wall properties, meta-analysis, cross-sectional compliance, lower limb arteries, common femoral artery, healthy adults

## Abstract

**Background/Objectives:** Arterial wall compliance is a key biomechanical property influencing local hemodynamics, vascular remodeling, and surgical performance. Despite its clinical relevance, reference values in lower limb arteries remain insufficiently characterized. This systematic review and meta-analysis aimed to synthesize existing evidence on cross-sectional compliance (CC) of the common femoral artery (CFA), superficial femoral artery (SFA), and popliteal artery (PA) in healthy adults. **Methods**: A systematic literature search was performed according to PRISMA guidelines to identify studies reporting arterial wall compliance in healthy adults. Study characteristics, measurement techniques, and compliance values were extracted. Risk of bias was assessed using a QUADAS-2/COSMIN-based tool. Pooled estimates were calculated using random effects meta-analysis, PROSPERO CRD420251208399. **Results**: Thirty-four studies with a total of 3452 participants were included. Reported compliance values varied substantially. The pooled mean CC of the CFA was 0.95 (95%-CI 0.83–1.06, I^2^ = 99.2%). Sex-specific analysis showed a pooled mean CC of 1.07 (95%-CI 0.87–1.28, I^2^ = 99.0%) in males and 0.90 (95%-CI 0.75–1.05, I^2^ = 83.6%) in females. Meta-regression of age, sex, and BMI did not significantly demonstrate association between these moderators and CC or explain observed heterogeneity. Extracted CC values of the SFA ranged from 0.17 to 0.50 mm^2^/kPa, and mean CC values of the PA ranged from 0.44 to 0.62 mm^2^/kPa. **Conclusions**: Available data on CC in lower limb arteries are limited and heterogeneous. Given the substantial between-study heterogeneity, the pooled estimates resulting from the meta-analysis should therefore be considered descriptive and should not be interpreted as definitive physiological reference values.

## 1. Introduction

The arterial wall is a highly specialized biological structure that continuously adapts to pulsatile hemodynamic forces throughout the cardiac cycle. Unlike rigid conduits, the arterial wall exhibits the ability to expand and recoil in response to cyclic pressure changes, a property referred to as arterial wall compliance. This non-linear characteristic reflects the pressure-dependent interaction of elastin and collagen fibers within the vessel wall. While elastin dominates vessel behavior at lower pressures, progressive recruitment of collagen fibers with increasing pressure leads to reduced elasticity and increased arterial stiffness [1]. Mechanically, cross-sectional compliance (CC) is defined as the change in arterial cross-sectional area (ΔA) per change in pressure (ΔP). Accordingly, CC can be expressed as

CC = ∆A/∆P = π/4 × (D_s^2^ − D_d^2^)/∆P

where Ds and Dd denote the systolic and diastolic arterial diameters [1,2].

Arterial CC can be assessed using both in vitro and in vivo approaches. In vitro testing of excised vessel segments allows precise biomechanical characterization under controlled conditions whereas in vivo compliance can be assessed non-invasively using ultrasound, applanation tonometry, or magnetic resonance imaging (MRI), typically in combination with blood pressure measurements [2,3].

Alterations in arterial CC have been associated with various conditions such as aging [4], smoking [5], arterial hypertension and diabetes mellitus [6]. Understanding these alterations and the associated hemodynamic changes is crucial for the interpretation and treatment of vascular diseases. For instance, a compliance mismatch commonly occurring at the anastomotic sites of synthetic bypass grafts to native vessels is a recognized driver of vascular complications, including intimal hyperplasia and aneurysm formation at the anastomosis. Such complications are driven by altered flow patterns and associated changes in local wall shear stress [7,8,9]. Autologous vein bypass grafts generally demonstrate superior patency rates compared with synthetic grafts and are therefore considered the preferred conduit for lower-extremity revascularization [10,11,12]. However, suitable autologous vein material is unavailable in a substantial proportion of patients, for example, due to varicose degeneration, thrombotic damage, or previous use during other surgical procedures. Allogeneic or xenogeneic vascular grafts remain of limited practical use due to restricted availability and logistical challenges, meaning that synthetic vascular grafts are predominantly used in routine clinical practice [12,13,14,15].

Synthetic grafts with a CC similar to that of native vessels could potentially reduce complications associated with compliance mismatch and improve patency rates. Therefore, understanding the compliance of lower-extremity arteries and defining robust reference values remain essential.

In previous research, peripheral arterial compliance of the lower limb has been measured and estimated multiple times. However, no fixed reference values for lower limb CC in humans without vascular disease have yet been established in the literature. Therefore, the aim of this systematic review is to collect, evaluate, and synthesize data from previous studies to explore the extent to which the existing data permit quantitative synthesis and the establishment of a reference framework.

## 2. Materials and Methods

### 2.1. Protocol and Review Design

The review protocol was developed and registered in advance in the PROSPERO database (registration number: CRD420251208399). The systematic review was designed and conducted in accordance with the Preferred Reporting Items for Systematic Reviews and Meta-Analyses (PRISMA) guidelines [16] and specific recommendations for systematic reviews in surgery [17].

### 2.2. Development of Inclusion Criteria

Inclusion criteria were defined using the PICOS framework (Population, Intervention, Comparison, Outcome, Study type) [18].

- Population: Healthy adult men and women without evidence of cardiovascular, metabolic, or vascular disease. Professional athletes were excluded.
- Intervention/Assessment: Non-invasive measurement methods used to assess arterial CC.
- Comparison: Not applicable.
- Outcome: CC or estimated CC of the common femoral artery (CFA), superficial femoral artery (SFA), and popliteal artery (PA).
- Study type: All study designs meeting the inclusion criteria were considered. Case reports and studies comparing two or more assessment methods were excluded.

### 2.3. Systematic Literature Search

A systematic literature search was conducted in the electronic databases MEDLINE, CENTRAL and Web of Science to identify studies investigating arterial wall compliance of lower limb arteries. Gray literature, conference proceedings, and doctoral dissertations were also considered. The search strategy was developed and performed in collaboration with a methodological expert (E.K.). The full search strategy is provided in the electronic Supplementary Materials (S1). The final database search was performed on 11 November 2025. Only studies published in English or German were included. Reference lists of all studies meeting the eligibility criteria were screened manually to identify additional relevant publications. When full texts were not available, corresponding authors were contacted where contact information was available.

### 2.4. Review Process and Study Selection

All records identified through the literature search were independently screened by two reviewers (C.B. & B.H.). In the first step, titles and abstracts were assessed for eligibility. In a second step, full texts of potentially relevant studies were reviewed against the predefined inclusion criteria. Disagreements were resolved by discussion or, if necessary, by consultation of a third reviewer (P.D.). Data extraction was performed by one reviewer using a predefined data extraction form and independently cross-checked by a second reviewer (C.B.). A third reviewer supervised the data extraction process (J.F.). In cases where multiple studies originated from the same center or reported data from overlapping participant cohorts, only the study with the largest sample size and the most comprehensive methodological description was included to avoid duplication of data.

### 2.5. Data Items

The following data items were extracted from all included studies: mean age ± standard deviation (SD) and/or age range, sex, body mass index (BMI, kg/m^2^; mean ± SD), smoking status (smokers included, excluded, or not assessed), and CC ± SD of the CFA, SFA, and PA. CC values were reported in mm^2^/kPa. When CC was originally reported in mm^2^/mmHg, values were converted to mm^2^/kPa using the inverse of the pressure conversion factor (1 mmHg = 0.133 kPa), i.e., by multiplying by 7.5006, to ensure consistent data synthesis across studies. If numerical data were presented only graphically, corresponding authors were contacted to request the original data where contact information was available. In cases where authors could not be contacted or did not respond, data were extracted from figures using the open-source software WebPlotDigitizer (version 5.2, Automeris LLC, Dublin, Ireland) [19]. To explore whether the heterogeneity observed for CC could be addressed by an alternative measure of arterial distensibility, a supplementary meta-analysis of distensibility coefficient (DC) values was performed. The analysis was restricted to studies included in the present systematic review for which DC data for the CFA was available. No additional literature search or studies were included specifically for DC analysis.

### 2.6. Risk of Bias Assessment

Risk of bias (RoB) was assessed using a purpose-built assessment tool developed specifically for this review, based on key domains of two established frameworks, QUADAS-2 (Quality Assessment of Diagnostic Accuracy Studies) [20] and COSMIN (Consensus-based Standards for the Selection of Health Measurement Instruments) [21].

This approach was chosen because no existing RoB assessment tool was fully suitable for the specific aims of this review, given the heterogeneity of study designs and measurement methods across the included studies. The assessment tool focuses on methodological aspects particularly relevant to this review, including characteristics of the study population as well as the validity and reliability of the applied measurement methods. Each domain was judged as having low, moderate, or high RoB. No overall RoB score was calculated, as the purpose of the assessment was to identify domain-specific methodological limitations rather than to rank studies by overall quality.

Before conducting the formal risk-of-bias assessment, the tool was initially applied to several included studies representing the different study designs and measurement approaches identified in the review. This initial application served to assess the applicability and clarity of the assessment criteria and to identify any ambiguities in their interpretation. Where necessary, minor clarifications were made before the final assessment was conducted for all included studies.

Risk of bias was assessed independently by two reviewers. Disagreements were resolved by consensus, with involvement of a third reviewer if necessary. The complete RoB assessment tool is provided in the Electronic Supplementary Materials (Table S1).

### 2.7. Effect Measures

The primary effect measure of this review was CC. Values were extracted as reported in the original studies but converted to a uniform unit or pooled if necessary to allow comparison and synthesis across studies.

### 2.8. Data Synthesis

Statistical analyses were performed using R Statistical Software (Version 4.5.2; R Core Team 2025) [22,23]. Meta-analyses and meta-regressions were conducted using the meta package in R [24,25]. Pooled estimates of CC were calculated using random effects meta-analysis to account for expected methodological and clinical heterogeneity between studies. Meta-analyses were conducted for arterial segments with data from at least seven studies. For arterial segments with fewer than seven studies, results were summarized descriptively and visualized using dot plots to illustrate individual study estimates. To further explore potential sources of heterogeneity, meta-regression analyses were performed to examine whether study-level characteristics were associated with variations in reported CC.

### 2.9. Assessment of Heterogeneity

Between-study heterogeneity was assessed using the I^2^ statistic and τ^2^, which quantify the proportion of total variability attributable to between-study differences and the estimated variance of true effect sizes, respectively. I^2^ values were interpreted according to commonly used thresholds, with 0–40% heterogeneity considered potentially unimportant, 30–60% may indicate moderate heterogeneity, 50–90% may indicate substantial heterogeneity and 75–100% considerable heterogeneity [26].

### 2.10. Assessment of Reporting Bias

Due to the heterogeneity of included study designs, measurement techniques and outcome, a quantitative assessment of reporting bias (e.g., funnel plot analysis) was not feasible for this systematic review. Therefore, reporting bias was assessed qualitatively by evaluating methods, reported results and the presence of selectively reported compliance parameters across included studies.

## 3. Results

**Figure 1:**
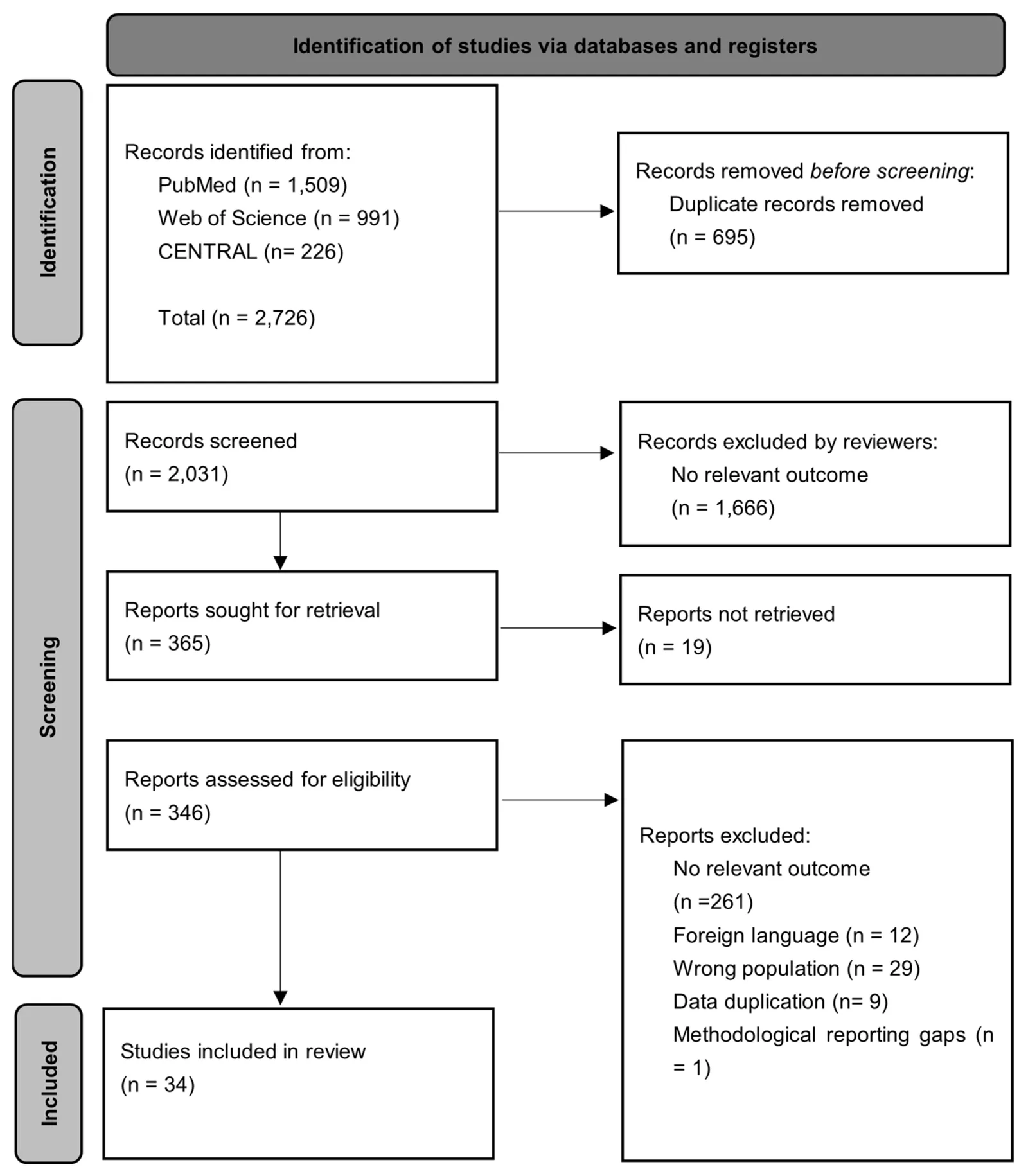
PRISMA flow diagram illustrating the study selection and screening process for the systematic review on cross-sectional arterial wall compliance of the common femoral, superficial femoral, and popliteal arteries in healthy adults. Source: [16].

The literature search identified 2726 records. After removal of duplicates, 2031 studies entered the screening process. Titles and abstracts were independently screened by two reviewers according to the predefined inclusion and exclusion criteria. Following full-text assessment, 34 studies were deemed eligible and included in this review [4,5,6,21,22,23,24,25,26,27,28,29,30,31,32,33,34,35,36,37,38,39,40,41,42,43,44,45,46,47,48,49,50,51] (Table 1). One study was excluded at the full-text stage due to insufficient methodological detail, including an undefined study population and unspecified measurement units. The full selection process can be followed in Figure 1.

Of the included studies, 29 assessed CC of the CFA [4,5,6,27,28,29,30,31,32,33,34,35,36,37,38,39,40,41,42,43,44,45,46,47,48,49,50,51,52], five assessed the SFA [53,54,55,56,57] and three assessed the PA [36,48,55]. Three studies reported measurements at more than one arterial site [36,48,55]. The majority of eligible studies (91.18%) used B-mode and/or M-mode ultrasound for compliance assessment. Four studies combined B-mode ultrasound with applanation tonometry [38,39,42,57], and two studies used other methods to assess the CC [32,51].

**Table 1:** Key demographic characteristics and main outcomes of the included studies.

Author, Year	Design	Participants (n)	Mean Age	Age Range	Female (%)	Smokers Included	Mean CC mm^2^/kPa (SD)		
							CFA	SFA	PA
Willekes et al., 1999 [27]	Controlled quasi-experimental study	14	n.a	18–25	100	n.a.	0.8 (0.4)		
Willekes et al., 1998 [28]	Controlled quasi-experimental study	24	n.a	18–35	100	n.a.	0.67 (0.4)		
Wijnen et al., 1991 [29]	Case–Control	15	24	n.a	0	no	1.43 (0.12)		
Vermeersch et al., 2008 [4]	Cohort study	2195	45.1	35–56	51.5	yes	0.76 (0.94)		
Vanmolkot et al., 2007 [30]	Case–Control	50	24.6	18–35	78	yes	1.42 (0.57)		
van Vonderen et al., 2009 [31]	Case–Control	52	49.6	n.a	0	yes	1.09 (0.05)		
van den Berkmortel et al., 2004 [5]	Case–Control	50	46	n.a	48	no	0.7 (0.35)		
van den Berkmortel et al., 1998 [32]	Cross-sectional study	13	33.4	n.a	54	no	0.71 (0.17)		
van Bussel et al., 2011 [33]	Cohort study	293	36.6	n.a	52.9	yes	0.51 (0.24)		
Thijssen et al., 2007 [53]	Controlled quasi-experimental study	8	70	n.a	0	no		0.17 (0.06)	
Smilde et al., 1998 [34]	Cross-sectional study	28	39.0	24–66	n.a.	no	0.7 (0.2)		
Schmidt-Trucksäss et al., 2000 [35]	Cross-sectional study	20	24.6	n.a	0	n.a.	0.92 (0.48)		
Rakobowchuk et al., 2011 [36]	Interventional study	15	20.6	n.a	47	no	0.88 (0.31)		0.46 (0.18)
Poelkens et al., 2007 [37]	Intervenional study	12	71	63–90	0	no	1.09 (0.19)		
Miyachi et al., 2004 [38]	Randomized intervention study	28	22	20–38	0	no	0.71 (0.08)		
Miyachi et al., 2003 [39]	Cross-sectional study	32	38.3	20–60	0	no	0.68 (0.12)		
Luik et al., 1997 [40]	Case–Control	24	65.8	n.a	25	n.a.	0.67 (0.38)		
Liu et al., 2011 [54]	Case–Control	25	46.2	n.a	88	no		0.26 (0.11)	
Liu et al., 2011 [41]	Interventional study	21	21.3	19–23	0	no	0.67 (0.35)		
Lin et al., 2021 [42]	Interventional study	34	68.1	n.a	52.9	no	1.29 (0.23)		
Lambert et al., 1997 [43]	Interventional study	32	24.8	n.a		yes	1.02 (0.36)		
Kool et al., 1995 [6]	Case–Control	30	26	n.a	36.7	yes	0.94 (0.41)		
Kılıçaslan et al., 2023 [44]	Case–Control	39	35.1	n.a	54	yes	1.28 (0.68)		
Gonzales et al., 2014 [55]	Interventional study	48	22	18–31	50	n.a.		0.48 (0.17)	
Gonzales et al., 2013 [45]	Cross-sectional study	21	68	61–78	0	n.a.	1.61 (0.8)		0.62 (0.32)
Giltay et al., 1999 [46]	Cross-sectional study	34	25.5	n.a	50	yes	0.87 (0.26)		
Fabry et al., 2011 [47]	Randomized double-blind cross-over	20	25	n.a	100	yes	1.09 (0.56)		
de Groot et al., 2005 [56]	Case–Control	8	41	n.a	50	n.a.		0.5 (0.13)	
De Basso et al., 2015 [48]	Case–Control	66	60.4	40–80	56.1	no	1.17 (0.30)		
Credeur et al., 2018 [49]	Interventional study	20	46	n.a	n.a.	n.a.	0.45 (0.08)		0.44 (0.16)
Brunt et al., 2016 [57]	Interventional study	10	21.9	18–30	60	no		0.45 (0.08)	
Baykara et al., 2017 [50]	Interventional study	38	26.5	18–40	16	n.a.	0.9 (0.05)		
Armentano et al., 1995 [51]	Cross-sectional case–control	16	48	n.a	0	yes	1.69 (0.34)		
Arcaro et al., 2004 [52]	Interventional study	12	n.a	21–37	0	no	1.24		

CC = cross-sectional compliance; CFA = common femoral artery; SFA = superficial femoral artery; PA = popliteal artery; SD = standard deviation; n.a. = not assessed.

### 3.1. Cross-Sectional Compliance of the Common Femoral Artery

**Figure 2:**
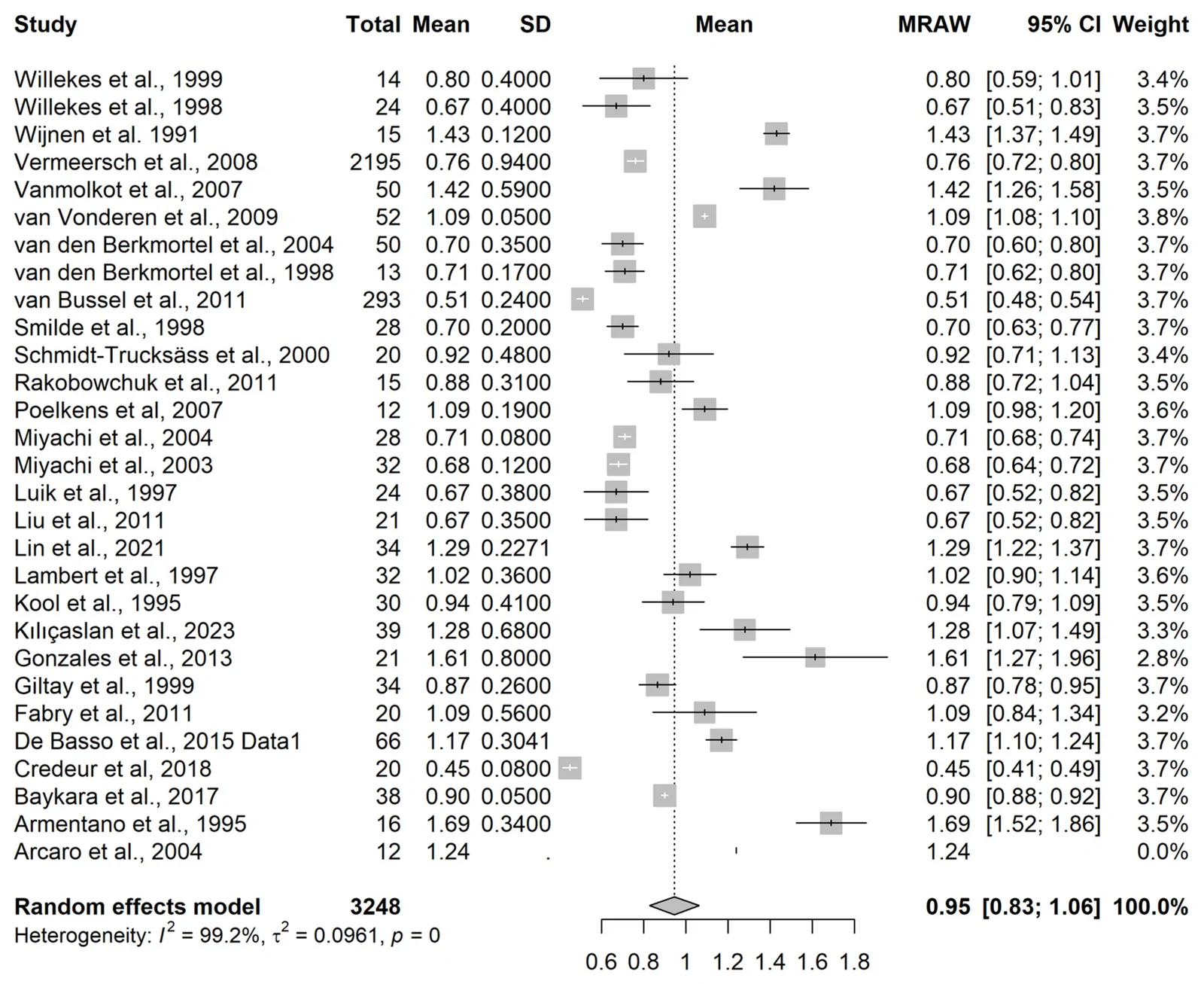
Meta-analysis of mean cross-sectional compliance of the common femoral artery [4,5,6,27,28,29,30,31,32,33,34,35,36,37,38,39,40,41,42,43,44,45,46,47,48,49,50,51,52]. Total = number of participants included per study; SD = standard deviation; MRAW = untransformed mean included in calculating overall mean; CI = confidence interval; The vertical line represents the overall mean of all studies.

CC of the CFA was assessed in 3248 participants in total. The mean CC of the CFA ranges between 0.67 and 1.69 mm^2^/kPa. Using the random effects model, the overall mean CC of the CFA was 0.95 (95%-CI 0.83–1.06). Heterogeneity assessment showed an I^2^ value of 99.2% (Figure 2). For the supplementary meta-analysis of DC of the CFA, values from 17 included studies were available. One study was excluded from meta-analysis because the scale of the reported data could not be reliably determined from the presented figure. The pooled mean DC was 15.877 (95%-CI 12.800–18.954), with substantial between-study heterogeneity (I^2^ = 99.7%), comparable to that observed for CC (Figure S1, Supplementary Materials).

### 3.2. Subgroup Analysis

**Figure 3:**
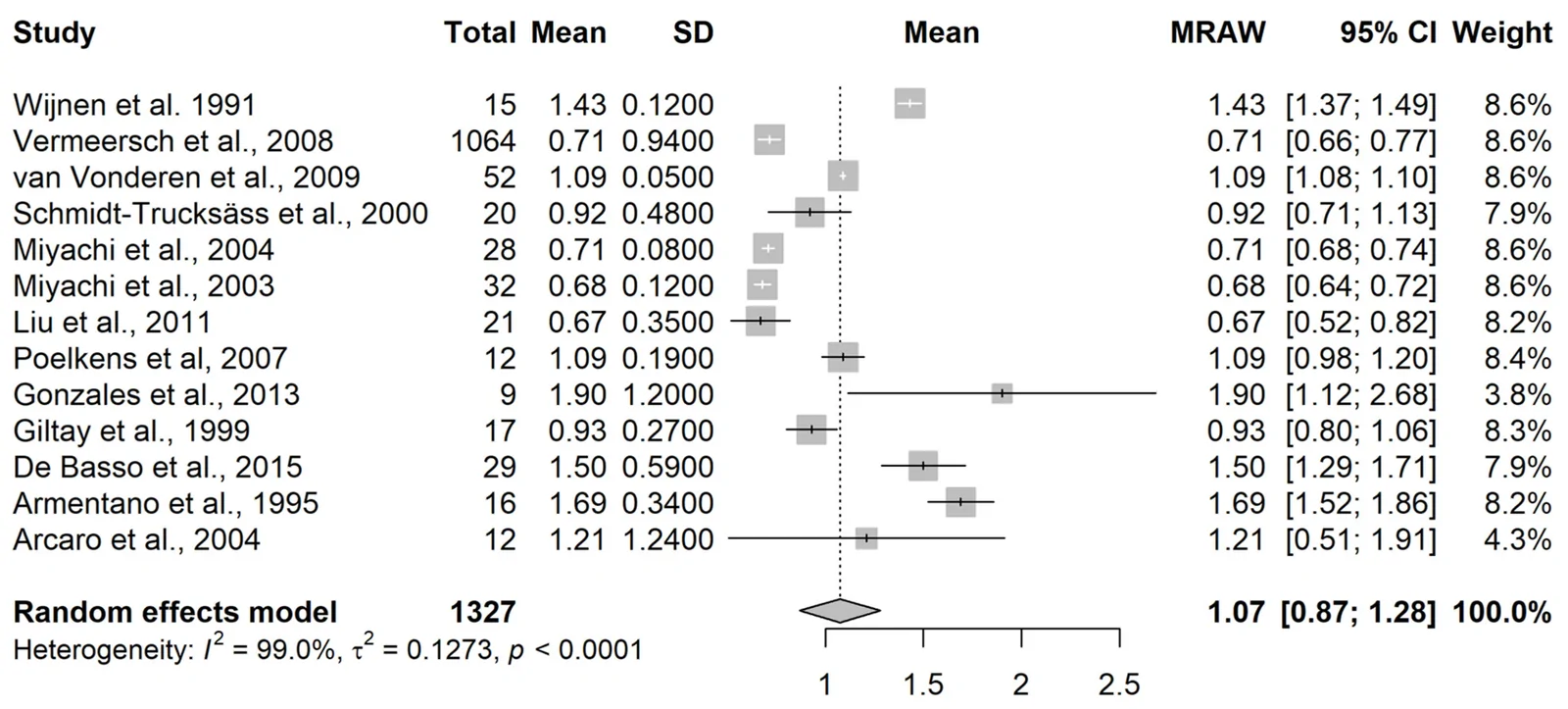
Subgroup analysis of studies providing data for the mean cross-sectional compliance of the common femoral artery in male participants [4,29,31,35,37,38,39,41,45,46,48,51,52]. Total = number of participants included per study; SD = standard deviation; MRAW = untransformed mean included in calculating overall mean; CI = confidence interval. The vertical line represents the overall mean of all studies.

**Figure 4:**
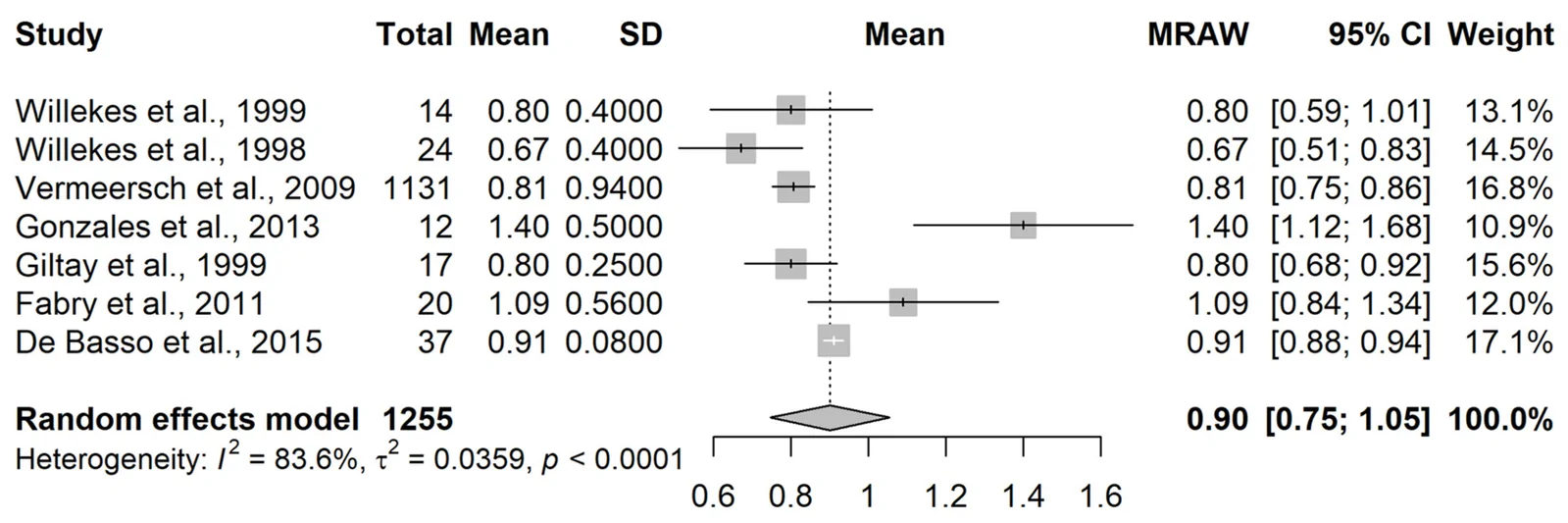
Subgroup analysis of studies providing data for the mean cross-sectional compliance of the common femoral artery in female participants [4,27,28,45,46,47,48]. Total = number of participants included per study; SD = standard deviation; MRAW = untransformed mean included in calculating overall mean; CI = confidence interval. The vertical line represents the overall mean of all studies.

A sex-specific subgroup analysis was performed for the CFA. Sixteen studies were eligible for this analysis. Of these, seven studies reported separate male and female cohorts, whereas nine studies included either exclusively male or exclusively female cohorts. In total, 2558 participants were included in the subgroup analysis, comprising 1327 men and 1231 women. Mean CC of the CFA in males ranged from 0.67 to 1.90 mm^2^/kPa. Using the random effects model, the overall mean CC of the CFA in male participants was 1.07 (95%-CI 0.87–1.28). with a heterogeneity of I^2^ = 99.0% (Figure 3). In females mean CC of the CFA ranged from 0.67 to 1.40 mm^2^/kPa. Using the random effects model, overall mean CC of the CFA in females was 0.90 (95%-CI 0.75–1.05). Heterogeneity assessment showed an I^2^ value of 83.6% (Figure 4).

### 3.3. Meta-Regression Analysis

Meta-regression analyses were performed to investigate whether mean participant age, proportion of female participants, and body mass index (BMI) were associated with CFA compliance. Data on mean age was available for 26 studies. Data on the proportion of female participants were available for 26 studies; however, one study lacked a corresponding standard deviation for CFA compliance and was therefore excluded from the meta-regression, resulting in 25 eligible studies. BMI data was available for 20 studies, of which 19 could be included in the meta-regression because one study lacked a corresponding standard deviation for CFA compliance.

Substantial residual heterogeneity remained for all moderator analyses (age: I^2^ = 99.34%; female proportion: I^2^ = 99.36%; BMI: I^2^ = 99.54%). Significant residual heterogeneity was observed after inclusion of mean age (QE = 2812.79, *p* < 0.001), female proportion (QE = 1859.68, *p* < 0.001), and BMI (QE = 2509.50, *p* < 0.001). None of the investigated moderators were significantly associated with CFA compliance. Mean participant age showed a small positive, but non-significant association with compliance (β = 0.0040, 95% CI −0.0038 to 0.0118; QM = 1.007, *p* = 0.316) (Figure S2 Supplementary Materials). Similarly, the proportion of female participants was not significantly associated with compliance (β = −0.0005, 95% CI −0.0044 to 0.0033; QM = 0.069, *p* = 0.793) (Figure S3, Supplementary Materials), nor was BMI (β = −0.0119, 95% CI −0.0913 to 0.0674; QM = 0.087, *p* = 0.768) (Figure S4, Supplementary Materials). The corresponding meta-regression models explained none of the observed between-study heterogeneity (R^2^ = 0% for all three moderators). A reliable subgroup or meta-regression analysis for smoking status was not feasible because of insufficient data.

### 3.4. Cross-Sectional Compliance of the Superficial Femoral Artery and Popliteal Artery

**Figure 5:**
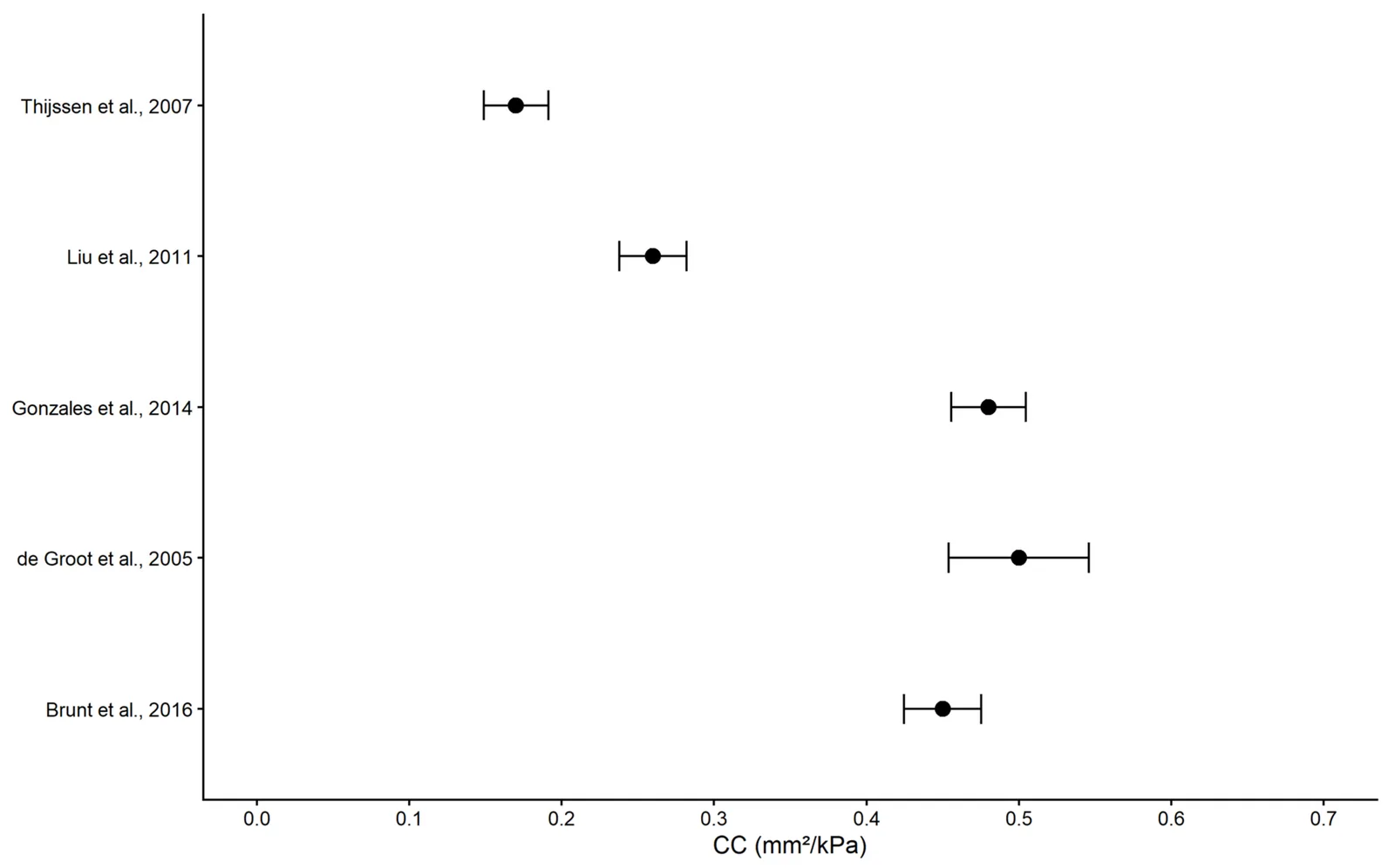
Dot plot of mean cross-sectional compliance (mm^2^/kPa) of the superficial femoral artery with corresponding standard deviations for each included study [53,54,55,56,57]. CC = cross-sectional compliance.

**Figure 6:**
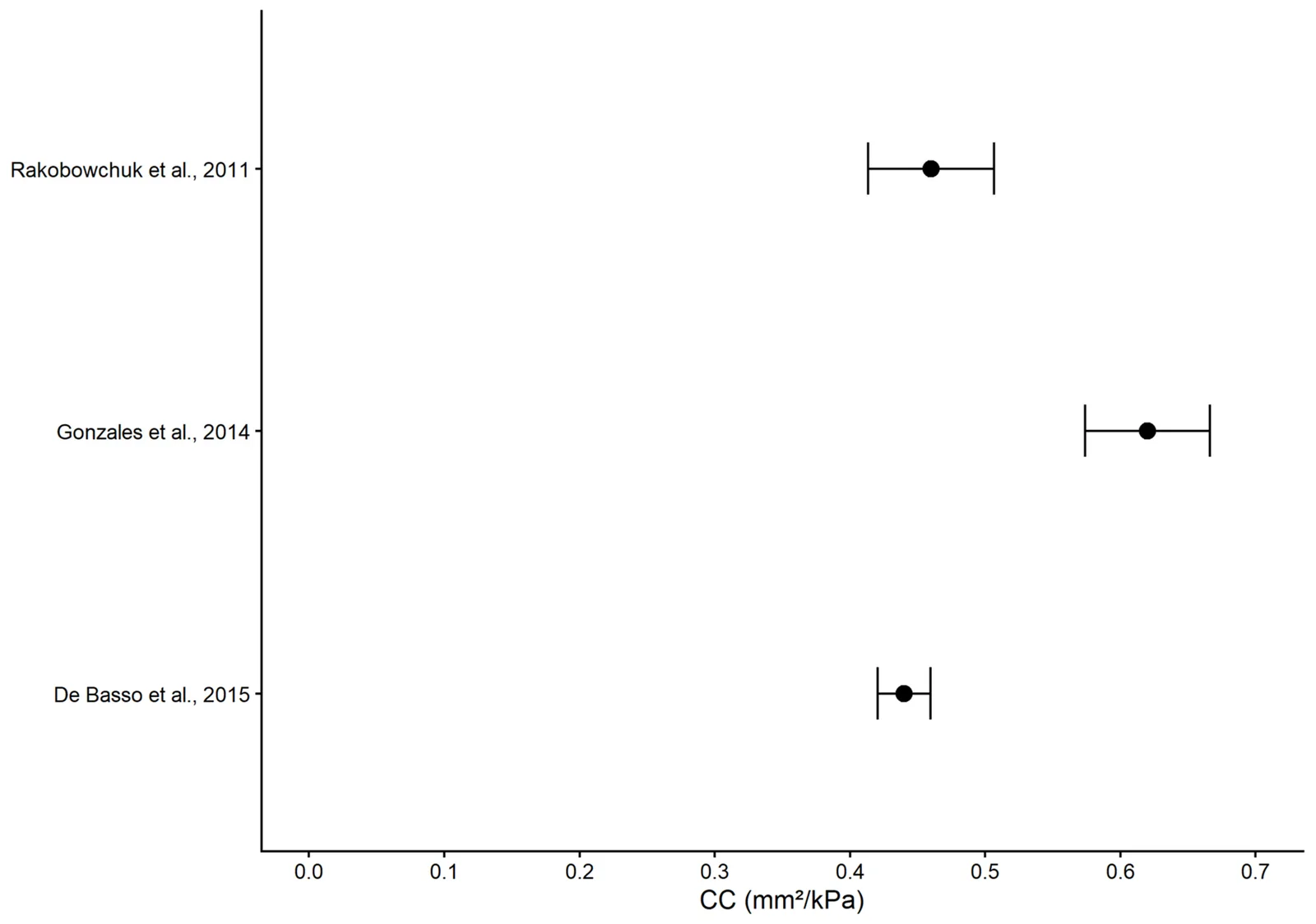
Dot plot of mean cross-sectional compliance (mm^2^/kPa) of the popliteal artery with corresponding standard deviations for each included study [36,48,55]. CC = cross-sectional compliance.

Due to the limited number of studies investigating the SFA (*n* = 5) and the PA (*n* = 3), no quantitative synthesis was performed. Instead, extracted study-level CC values are presented descriptively. CC of the SFA was assessed in 99 participants and CC of the PA was assessed in 129 participants. The reported mean CC values of the SFA ranged from 0.17 to 0.50 mm^2^/kPa, whereas mean CC values of the PA ranged from 0.44 to 0.62 mm^2^/kPa (Figure 5 and Figure 6).

### 3.5. Risk of Bias

The conducted assessment of RoB focused on measurement procedures, measurement reliability, and study design characteristics across the included studies.

According to RoB assessment, 27 out of the 34 included studies described the measurement techniques used sufficiently. In the majority of studies (91.18%), the formula used to calculate CC is reported in the Methods section. Regarding measurement bias, three studies were rated as high risk because arterial compliance was not clearly defined. Four studies were rated as having a moderate risk of measurement bias, including three studies with insufficient methodological detail and one study applying a substantially different measurement approach. In two studies, the femoral artery segment assessed was not explicitly specified, resulting in uncertainty as to whether the common or superficial femoral artery was examined. In the domain of measurement reliability, nine studies were rated as moderate risk because no explicit information on reliability of their methods was provided. All remaining studies were rated as low risk in this category. Selection bias and confounding were the domains most frequently rated with elevated risk of bias. Fifteen studies were rated as having a moderate risk of selection bias due to insufficiently defined inclusion criteria for ‘healthy adults,’ and one study was rated as high risk. Incomplete reporting of participant characteristics such as sex distribution, body mass index, or participants’ smoking status contributed to moderate risk ratings for confounding in several studies. Two studies included fewer than ten participants and were therefore rated as high risk due to small sample size. Seven studies reported relevant data primarily in graphical form and have been rated with moderate RoB in the category reporting bias. No study was excluded from the review based on the RoB. The complete RoB assessment is provided in the Supplementary Materials (Table S2).

## 4. Discussion

This systematic review aimed to synthesize available evidence on CC of the lower limb in healthy adults. Despite the identification of a considerable number of studies reporting arterial compliance, the available evidence did not provide a sufficiently consistent basis for establishing reliable physiological reference values. The reported pooled mean CC of 0.95 should therefore be regarded as a descriptive summary of the available evidence rather than as a physiological reference value. In particular, substantial heterogeneity was observed among studies investigating the CFA, while evidence for arterial segments distal to the CFA remained limited. During the review process, several important methodological and conceptual challenges of current evidence on the topic became apparent.

Most notably, the term ‘compliance’ was frequently used as an umbrella term for different arterial wall properties rather than as a strictly defined physical parameter. In several studies, compliance was used interchangeably with related measures such as vascular strain, arterial distensibility, or other stiffness indices (e.g., stiffness index β), although these parameters represent distinct biomechanical concepts (Table S3). This inconsistent use of terminology substantially limited the comparability of results across studies and reduced the number of studies eligible for quantitative synthesis. Thus, even before quantitative pooling, considerable conceptual heterogeneity was present in the literature.

Among the studies in which CC was clearly defined, the meta-analysis of the CFA revealed extremely high heterogeneity that could not be explained by the moderator variables examined in the meta-regression analyses. Because CC represents the absolute change in arterial cross-sectional area per unit pressure change, it is inherently influenced by baseline arterial caliber. To assess whether differences in arterial size could contribute to the observed heterogeneity, we additionally performed a meta-analysis of DC, which normalizes the change in cross-sectional area to the baseline arterial area. Despite this normalization, the DC meta-analysis showed similarly substantial between-study heterogeneity compared with CC. Thus, normalization for baseline arterial caliber did not materially reduce the observed heterogeneity, suggesting that differences in arterial size alone are unlikely to sufficiently explain the substantial between-study variability. One possible explanation is the presence of confounders that were not consistently assessed or reported in the included studies. For example, participants’ smoking status could not be evaluated in the present analysis due to inconsistent reporting, although it may plausibly influence arterial wall properties. This interpretation is supported by the RoB, which indicated that the highest potential for bias occurred in the domains of selection bias and confounding. In a considerable proportion of studies, the criteria used to define ‘healthy adults’ were reported only with limited detail, and relevant participant characteristics were not consistently documented. As these factors may influence arterial wall mechanics, incomplete characterization of study populations may have contributed to the substantial between-study variability observed in the present analysis.

Interestingly, the moderator variables ‘age’ and ‘female’ did not significantly explain the observed heterogeneity, although age- and gender-related reductions in arterial compliance have been reported in previous studies [4,58,59,60,61]. The absence of a significant association between age and CC and between female proportion and CC in the present meta-regression should therefore be interpreted cautiously. The cross-sectional nature of the included studies does not allow longitudinal changes in arterial compliance to be directly assessed, while differences in measurement devices, wall-tracking algorithms, measurement protocols, and the definition of a ‘healthy’ population may have obscured an underlying biological association. Accordingly, the present findings should not be interpreted as evidence against age- and sex-related declines in arterial compliance. Rather, they are consistent with previous studies demonstrating age-related alterations in arterial compliance, while suggesting that the magnitude and detectability of these associations may depend on the arterial segment, measurement methodology, and population characteristics.

In addition to known but insufficiently reported confounders, the influence of further, less obvious factors cannot be excluded. Experimental evidence suggests that even physiological conditions such as bladder filling may affect CC of the CFA 22. Such observations illustrate that additional, currently unrecognized confounders may contribute to variability in reported compliance values and further complicate CC between studies. Beyond conventional cardiovascular and metabolic factors, neurological disorders, autonomic dysfunction, and peripheral neuropathies may also potentially influence vascular tone and arterial compliance. However, these conditions were not consistently assessed or reported across the included studies. Their potential presence therefore represents an additional source of uncertainty when interpreting the pooled estimates as reference values for healthy adults. This is particularly relevant because autonomic regulation can affect vascular tone and arterial diameter at the time of measurement, potentially contributing to between-study differences independently of structural arterial wall properties.

Acute physiological and environmental conditions may represent a further source of variability. Factors such as ambient temperature, hydration status, recent caffeine or nicotine intake, physical activity before measurement, and medications affecting vascular tone or autonomic function may influence arterial diameter and vascular tone and, consequently, calculated CC. Such factors may alter the functional vascular state at the time of measurement without necessarily reflecting differences in intrinsic structural arterial wall properties. However, these factors were inconsistently reported across the included studies and could therefore not be systematically evaluated.

Regarding the RoB assessment, the overall risk of measurement and measurement reliability bias within individual studies appeared relatively low. Most studies applied established imaging-based techniques and reported the formula used to calculate CC. In studies where measurement reliability had not been explicitly validated, repeated measurements and averaging were commonly employed, which likely improved robustness. Nevertheless, incomplete methodological reporting in a small subset of studies introduces some uncertainty regarding the reproducibility of individual measurements.

Importantly, a relatively low risk of measurement bias within individual studies does not necessarily imply high comparability between studies. Another methodological challenge concerned anatomical specification. In two studies the term ‘femoral artery’ was not clearly defined, leaving uncertainty as to whether the CFA or SFA had been examined. Due to the similarity of the reported CC values to those observed in studies explicitly investigating the CFA, these measurements were assigned to the CFA group for data synthesis. While this approach maximized data utilization and maintained anatomical plausibility, a certain degree of misclassification cannot be entirely excluded.

Importantly, even if the RoB within individual studies appears relatively low, substantial variability may still arise between studies due to methodological differences. Potential contributors include differences in ultrasound equipment, wall-tracking algorithms, measurement protocols, and operator experience. Differences in the measurement of arterial diameter and blood pressure, as well as in participant preparation and measurement conditions, may further contribute to between-study variability. Furthermore, the extraction of numerical values from graphical representations represents an additional potential source of bias. Although standardized digitization procedures were applied, this process may introduce minor inaccuracies and therefore constitutes an additional limitation of the data synthesis. Taken together, these factors suggest that the observed heterogeneity may reflect a combination of population characteristics, physiological state, and measurement methodology rather than a single underlying source of variability.

Given the substantial heterogeneity and the limited number of studies per analysis, formal sensitivity analyses were not performed, as they were unlikely to provide meaningful additional insights beyond the conducted meta-regression.

Finally, the available evidence for arterial segments distal to the CFA remains extremely limited. Studies investigating the SFA and the PA were scarce. This limitation may be particularly relevant in the context of vascular reconstruction, where compliance mismatch between native vessels and synthetic bypass grafts, especially at distal anastomotic sites, is considered a key determinant of graft performance.

### 4.1. Reporting Bias

The assessment of CC in healthy adults was often not defined as a primary outcome, which may have contributed to incomplete reporting of relevant parameters. Moreover, only a limited number of interventional studies reporting non-significant findings was identified. Therefore, reporting bias cannot be fully excluded and should be considered a potential limitation of this review. A further limitation is the exclusion of potentially relevant studies due to unavailable full-text versions (Figure 1), which predominantly affected publications from the 1980s and early 1990s, a period during which much of the early research on CC and arterial wall properties was conducted.

Taken together, the available evidence suggests that the current literature does not yet provide a sufficiently consistent basis to define reliable physiological reference values for CC in the lower limb.

### 4.2. Future Perspective

To address these limitations and the given heterogeneity in the literature, future studies should aim to establish standardized reference conditions and measurement protocols for the assessment of lower limb arterial biomechanical properties. This should include clearly defined biomechanical parameters and standardized calculation methods for CC and related measures such as DC and strain, as well as consistent reporting of arterial diameter, blood pressure, measurement device, and wall-tracking methodology. In addition, participant preparation and relevant physiological factors should be considered and reported. The definition of a ‘healthy’ population should also be standardized and extend beyond the exclusion of cardiovascular and metabolic diseases to explicitly consider neurological conditions and autonomic dysfunction, which may affect vascular tone and arterial compliance. Moreover, a systematic assessment of DC and strain may provide complementary insights into the mechanical properties of lower limb arteries. Such methodological standardization and comprehensive assessment of arterial biomechanical properties would facilitate meaningful comparison between studies and may ultimately enable the establishment of clinically useful reference ranges.

## 5. Conclusions

This systematic review summarizes the current evidence on arterial compliance of the lower limb in healthy adults. Although the aim was to establish a reference framework, the available literature does not yet allow reliable physiological reference values to be defined across the lower limb arterial tree. Substantial heterogeneity, inconsistent terminology, and limited data for distal arterial segments highlight the need for standardized methodologies and clearer biomechanical definitions in future studies.

## Data Availability

The data presented in this study are available in the Supplementary Materials.

## Supplementary Materials

The following supporting information can be downloaded at https://www.mdpi.com/article/10.3390/jcm15176756/s1, S1: Search strategy, Table S1: Risk of bias assessment tool—key characteristics for each domain, Table S2: Risk of bias assessment, Figure S1: Meta-analysis of mean distensibility coefficient (DC) 10^−3^ kPa^−1^ of the common femoral artery, Figure S2: Bubble plot of the meta-regression between cross-sectional compliance of the common femoral artery and mean age of participants, Figure S3: Bubble plot of the meta-regression between cross-sectional compliance of the common femoral artery and percentage of female participants, Figure S4: Bubble plot of the meta-regression between cross-sectional compliance of the common femoral artery and mean body mass index (BMI) of participants, Table S3: Physical characteristics of arterial wall properties, Table S4: Demographic characteristics and extracted study data for all included studies, Table S5: Demographic characteristics and extracted study data for studies included in the male subgroup analysis, Table S6: Demographic characteristics and extracted study data for studies included in the female subgroup analysis.

## Abbreviations

The following abbreviations are used in this manuscript:

BMI	Body mass index
CC	Cross-sectional compliance
CFA	Common femoral artery
MRI	Magnetic resonance imaging
PA	Popliteal artery
RoB	Risk of bias
SD	Standard deviation
SFA	Superficial femoral artery
