# Supplementary material for: Arterial Wall Compliance of the Lower Limb Arteries in Healthy Adults—A Systematic Review and Meta-Analysis
Source: J Clin Med. 2026 Aug 31;15(17):6756. doi: 10.3390/jcm15176756 (PMC13566327; doi:10.3390/jcm15176756)
Supplement: Supplementary file 1 [file jcm-15-06756-s001.zip › jcm-4480376-supplementary.pdf]

Supplementary material

S1 Search strategy

Pubmed: 1509 hits

(vascular[tiab] OR arterial[tiab] OR aortic[tiab] OR artery[tiab] OR arteries[tiab]) AND (compliance[tiab] OR “diameter change”[tiab] OR distensibility[tiab] OR elasticity[tiab] OR stiffness[tiab]) OR "Compliance"[Mesh]

AND

“femoral artery”[tiab] OR “femoral arteries”[tiab] OR femoris[tiab] OR femoralis[tiab] OR "arteria poplitea"[tiab] OR "popliteal artery"[tiab] OR "a.poplitea"[tiab] OR "Femoral Artery"[Mesh] OR "Popliteal Artery"[Mesh] OR femoropopliteal[tiab] OR (arter*[tiab] AND (legs[tiab] OR (lower[tiab] AND (limb[tiab] OR extremit*[tiab]))))

NOT (animals [mh] NOT humans [mh])

Central: 226 hits

((vascular OR arterial OR aortic OR artery OR arteries) NEAR (compliance OR “diameter change” OR distensibility OR elasticity OR stiffness)):ti,ab,kw

OR MeSH descriptor: [Compliance] explode all trees

AND

(“femoral artery” OR “femoral arteries” OR femoris OR femoralis OR "arteria poplitea" OR "popliteal artery" OR "a.poplitea" OR femoropopliteal):ti,ab,kw

OR (arter* NEAR (legs OR (lower NEAR (limb OR extremit*)))):ti,ab,kw

OR MeSH descriptor: [Femoral Artery] explode all trees OR MeSH descriptor: [Popliteal Artery] explode all trees

WoS: 991 hits

TS = ((vascular OR arterial OR aortic OR artery OR arteries) NEAR (compliance OR “diameter change” OR distensibility OR elasticity OR stiffness))

AND

TS= (“femoral artery” OR “femoral arteries” OR femoris OR femoralis OR "arteria poplitea" OR "popliteal artery" OR "a.poplitea" OR femoropopliteal) OR

TS = (arter* NEAR (legs OR (lower NEAR (limb OR extremit*))))

Total: 2726

Total after removal of duplicates: 2031

Risk of Bias Assessment

Table S1: Risk of bias assessment tool – Key characteristics for each Domain

Domain	**Key question**	**Low risk of bias**	**Moderate risk of bias**	**High risk of bias**
Selection bias	Is the study population clearly defined and representative of healthy adults?	Clear inclusion and exclusion criteria; absence of cardiovascular or metabolic disease explicitly stated	Population described as “healthy” without clear definition	Inclusion criteria unclear or vascular disease not excluded
Measurement bias	Is arterial compliance appropriately defined and measured?	Cross-sectional Compliance (CC) defined as =/≈ ΔA/ΔP; measurement technique and synchronization of pressure and diameter clearly described; validated method used	Partial description of measurement protocol or unclear synchronization	Compliance not defined
Measurement reliability	Is the measurement procedure reproducible and technically sound?	Repeated measurements, averaging, or device validation reported	Reliability not addressed	Unvalidated device or unclear measurement conditions
Confounding bias	Are relevant physiological confounders considered?	Age, sex, smoking status and BMI reported; standardized measurement conditions	Confounders partially reported	Major confounders not reported or controlled
Reporting bias	Are outcomes fully and transparently reported?	Numerical compliance values reported for all assessed vessel segments	Partial reporting or mixed numerical and graphical presentation	Outcomes reported only graphically or inconsistently between methods and results
Other bias	Are there additional sources of bias?	Adequate sample size; no evidence of cohort overlap	Potential issues not fully addressed	Very small sample size, unclear device validation, or duplicate cohorts

**Table S2:** Risk of bias assessment								
**No.**	**Study**	**Selection bias**	**Measurement bias**	**Measurement reliability**	**Confounding bias**	**Reporting bias**	**Other bias**	**Comment**
1	Willekes et al., 1999	moderate	low	low	moderate	low	low	Linearized compliance approximation; smoking status not assessed; BMI not assessed
2	Willekes et al., 1998	moderate	low	low	moderate	low	low	Linearized compliance approximation; smoking status not assessed; BMI not assessed
3	Wijnen et al., 1991	moderate	low	low	moderate	low	low	Linearized compliance approximation; BMI not assessed
4	Vermeersch et al., 2008	moderate	low	moderate	moderate	moderate	low	BMI not assessed
5	Vanmolkot et al., 2007	low	low	low	low	low	low	
6	van Vonderen et al., 2009	moderate	low	low	low	low	low	
7	van den Berkmortel et al., 2004	low	high	low	low	low	low	CC not defined
8	van den Berkmortel et al., 1998	low	low	low	low	moderate	low	Linearized compliance approximation
9	van Bussel et al., 2011	moderate	low	moderate	low	low	low	
10	Thijssen et al., 2007	low	low	moderate	moderate	moderate	high	Linearized compliance approximation; BMI not assessed; small cohort size
11	Smilde et al., 1998	low	low	moderate	low	low	low	
12	Schmidt-Trucksäss et al., 2000	moderate	low	low	moderate	low	low	smoking status not assessed
13	Rakobowchuk et al., 2011	low	low	low	moderate	low	low	BMI not assessed
14	Poelkens et al., 2007	low	low	low	low	low	low	Linearized compliance approximation
15	Miyachi et al., 2004	low	low	low	moderate	low	low	BMI not assessed
16	Miyachi et al., 2003	low	high	low	moderate	low	low	CC not defined; BMI not assessed
17	Luik et al., 1997	moderate	moderate	low	moderate	low	low	measurement protocol not fully described; smoking status not assessed
18	Liu et al., 2011	low	low	low	low	low	low	
19	Liu et al., 2011	moderate	low	low	low	low	low	

20	Lin et al., 2021	low	low	low	low	moderate	low	Linearized compliance approximation
21	Lambert et al., 1997	moderate	moderate	low	low	moderate	low	Linearized compliance approximation; femoral artery not clearly defined
22	Kool et al., 1995	moderate	low	low	moderate	low	low	BMI not assessed
23	Kılıçaslan et al., 2023	low	moderate	moderate	low	low	low	femoral artery not clearly defined
24	Gonzales et al., 2014	moderate	low	moderate	moderate	low	low	smoking status not assessed
25	Gonzales et al., 2013	low	low	low	moderate	low	low	smoking status not assessed
26	Giltay et al., 1999	low	low	low	low	low	low	
27	Fabry et al., 2011	moderate	low	moderate	low	low	low	
28	de Groot et al., 2005	low	low	low	moderate	low	high	Linearized compliance approximation; BMI not assessed; smoking status not assessed: small cohort size
29	De Basso et al., 2015	low	low	low	moderate	moderate	low	
30	Credeur et al, 2018	high	low	low	moderate	low	low	exact female ratio unknown
31	Brunt et al., 2016	moderate	low	low	moderate	low	high	small cohort
32	Baykara et al., 2017	moderate	low	moderate	low	moderate	low	
33	Armentano et al., 1995	low	moderate	low	low	low	low	exclusive measurement method
34	Arcaro et al., 2004	low	high	moderate	low	low	low	CC not defined
CC = cross-sectional compliance; BMI = body mass index								

Additional meta-analysis of Distensibility coefficient (DC)

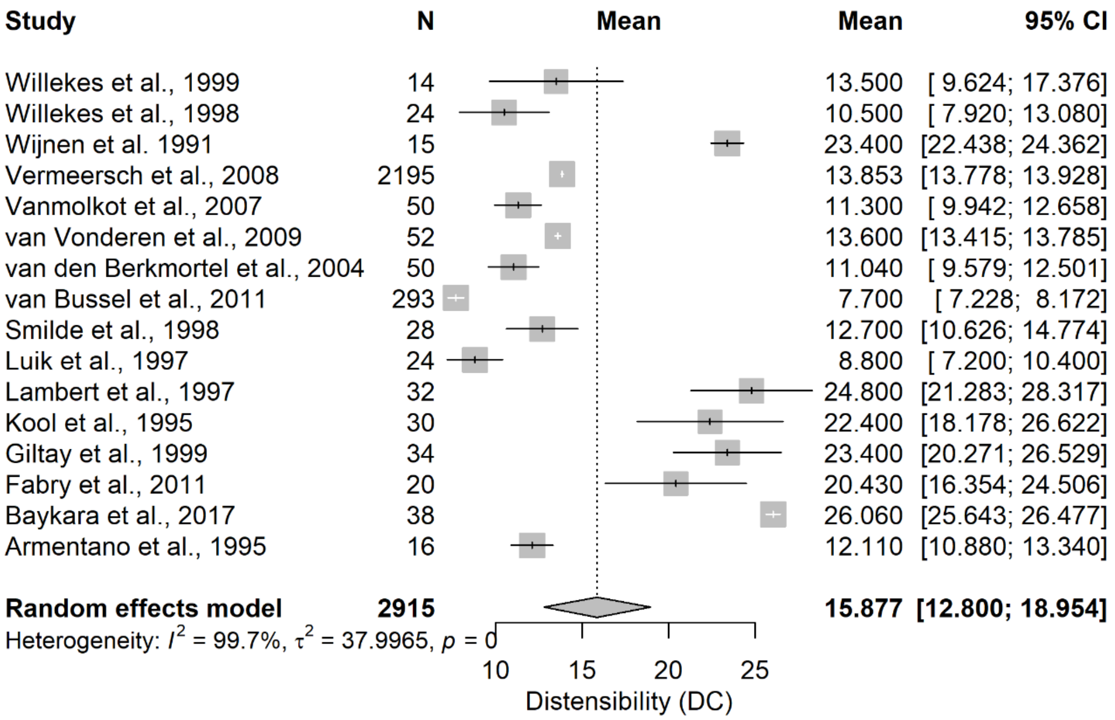

Figure S1 *Meta-analysis of mean distensibility coefficient (DC)$10^{-3}kPa^{-1}$ of the common femoral artery. n = number of participants included per study; CI = confidence interval; The vertical line represents the overall mean of all studies*

Meta-regression analysis

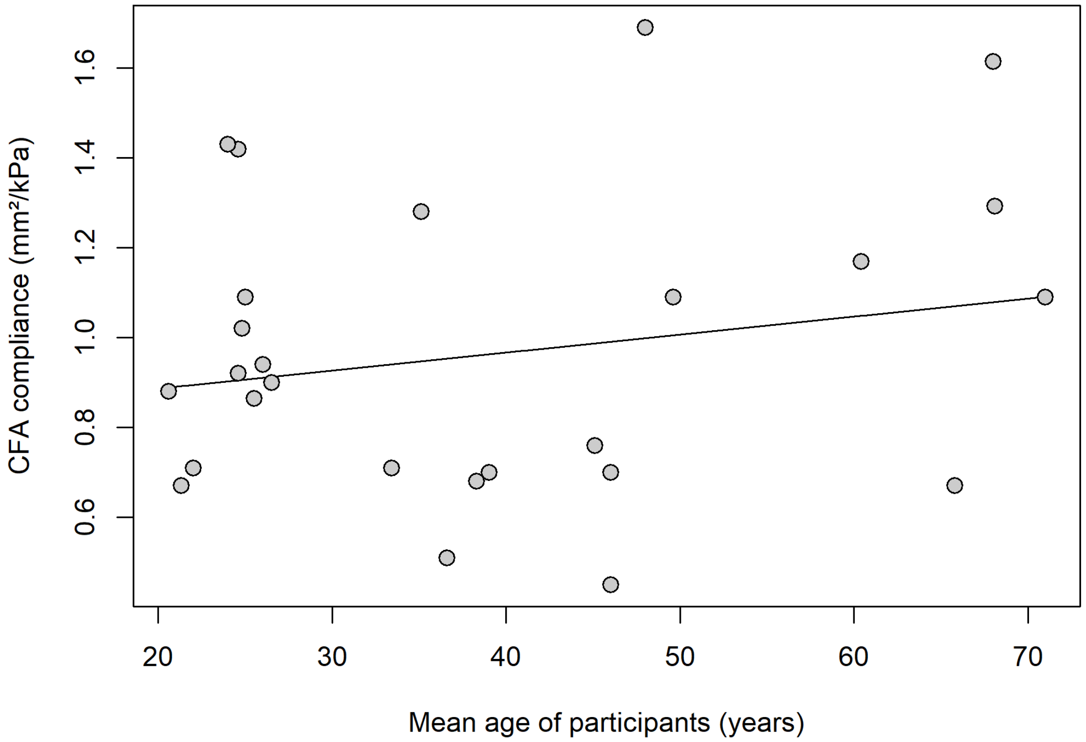

Figure S2 *Bubble plot of the meta-regression between cross-sectional compliance of the common femoral artery and mean age of participants*

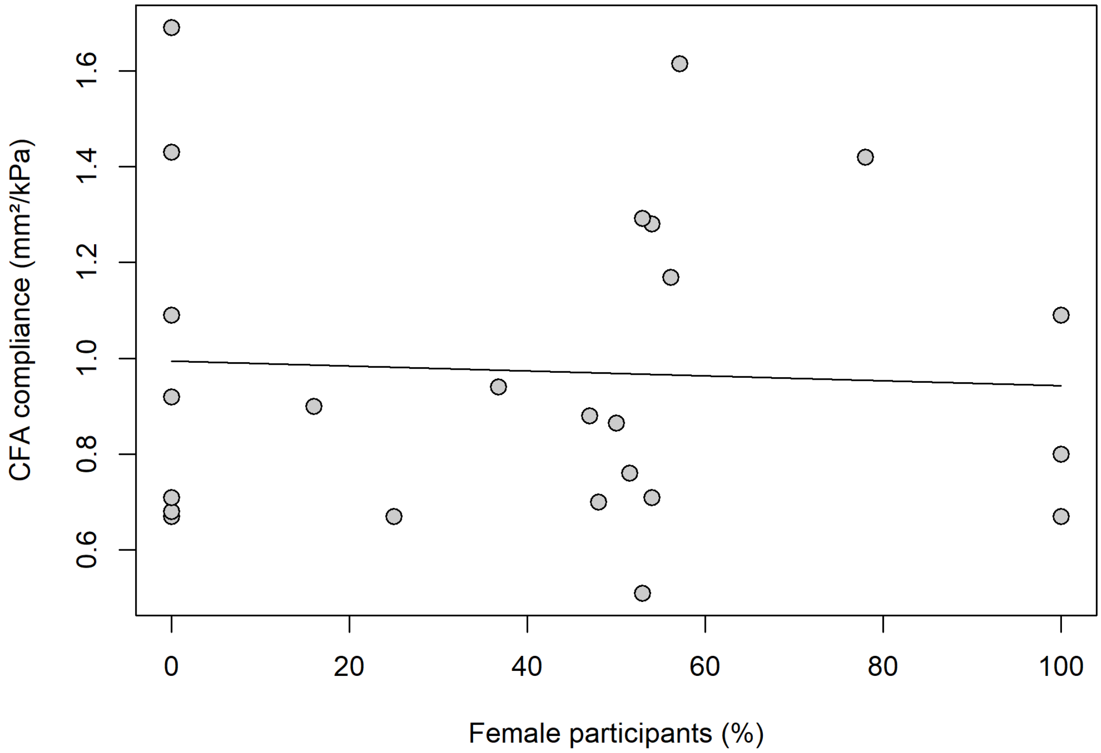

Figure S3 *Bubble plot of the meta-regression between cross-sectional compliance of the common femoral artery and percentage of female participants*

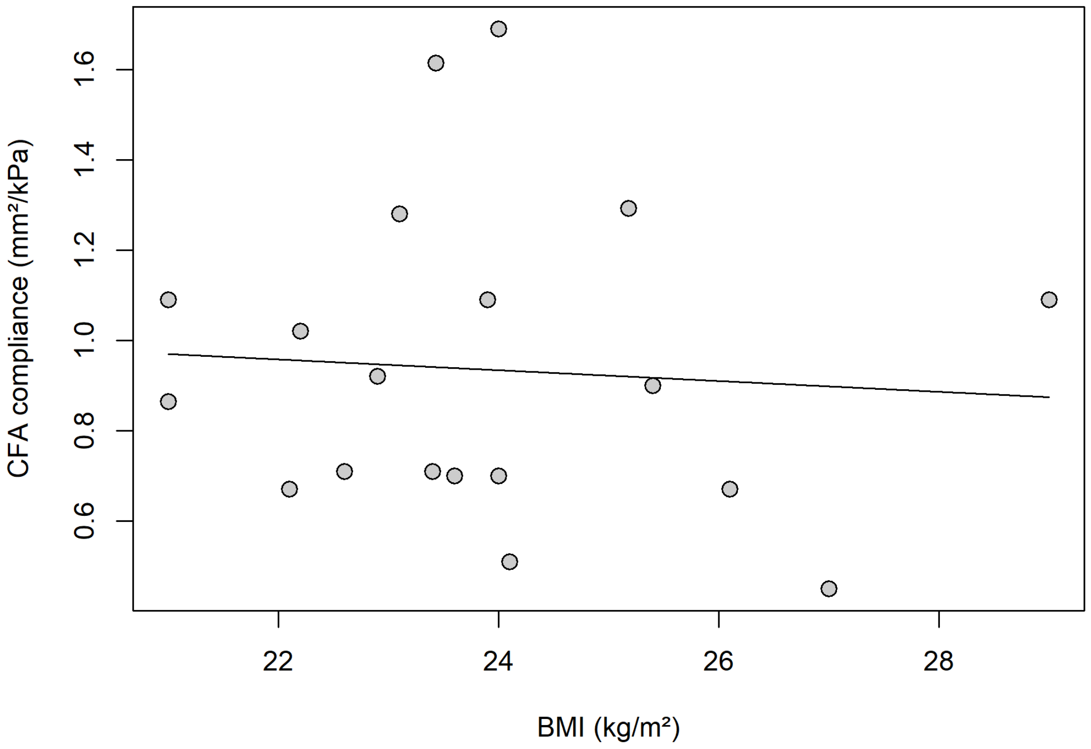

***Figure S4** Bubble plot of the meta-regression between cross-sectional compliance of the common femoral artery and mean body mass index (BMI) of participants*

Table S3: Physical characteristics of arterial wall properties

Parameter	Formula	Unit	Relationship to other parameters	Interpretation
**Strain**	$\frac{D_s - D_d}{D_d}$	dimensionless or %	Base geometric parameter	Relative arterial diameter change during the cardiac cycle
**Distensibility**	$\frac{(D_s - D_d)/D_d}{\Delta P}$	$mmHg^{-1}$ or $\%\cdot mmHg^{-1}$	Distensibility = Strain / ΔP	Relative diameter change normalized to pulse pressure
**Cross-sectional Compliance (CC)**	$\frac{\Delta A}{\Delta P}$	mm²/kPa	CC = Distensibility × A	Absolute change in arterial cross-sectional area per pressure change
**Stiffness Index β**	$\frac{\ln(P_s/P_d)}{(D_s - D_d)/D_d}$	dimensionless	Inversely related to strain	Pressure-independent index of arterial stiffness

D_s= systolic diameter; D_d= diastolic diameter; A= arterial cross-sectional area; P_s= systolic blood pressure; P_d= diastolic blood pressure; $\Delta P = P_s - P_d$;

Table S4: Demographic characteristics and extracted study data for all included studies

No.	Author et al., year	PMID	Journal name	Design	Participants (n)	Mean age	±SD age	Age range	Female (%)	BMI (mean)	±SD BMI	Smoking status	Measure-ment method	CC mm^2/kPa						DC 10^{-3} kPa^{-1}		converted units	pooled Data	Data extracted from Diagramm
														CFA		SFA		PA		CFA				
														mean	±SD	mean	±SD	mean	±SD	mean	±SD			
1	Willekes et al., 1999	10414888	Ultrasound Med Biol	Controlled quasi-experimental study	14			18-25	100			n.a	us	0.8	0.4					13.5	7.4			
2	Willekes et al., 1998	9740382	Ultrasound Med Biol	Controlled quasi-experimental study	24			18-35	100			n.a	us	0.67	0.4					10.5	6.45		x	
3	Wijnen et al. 1991	2036093	Basic Res Cardiol	Case Control	15	24	1		0			0	us	1.43	0.12					23.4	1.9			
4	Vermeersch et al., 2008	18551018	Jounal of Hypertension	Cohort study	2195	45.1	1.4	35–56	51.5			1	us	0.76	0.02					13.85	1.79		x	x
5	Vanmolkot et al., 2007	17460157	Neurology	Case Control	50	24.6	4	18-35	78			1	us	1.42	0.59					11.3	4.9			
6	van Vonderen et al., 2009	19131894	J Acquir Immune Defic Syndr	Case Control	52	49.6			0	23.9		1	us	1.09	0.05					13.6	0.68			
7	van den Berkmortel et al., 2004	15554598	Neth J Med	Case Control	50	46	11		48	23.6	2.5	0	us	0.7	0.35					11.04	5.27			
8	van den Berkmortel et al., 1998	9705034	J Hum Hypertens	Cross sectional study	13	33.4	8.7		54	23.4	2	0	other	0.71	0.17									x
9	van Bussel et al., 2011	21859964	Hypertension	Cohort study	293	36.6	0.6		52.9	24.1	3.2	1	us	0.51	0.24					7.7	4.12			
10	Thijssen et al., 2007	17394568	Acta Physiol (Oxf)	Controlled quasi-experimental study	8	70	1		0			0	us			0.17	0.06					x		x

11	Smilde et al., 1998	9848890	Arterioscler Thromb Vasc Biol	cross-sectional study	28	39	11	24–66	n.a.	24	1.6	0	us	0.7	0.2					12.7	5.6			
12	Schmidt-Trucksäss et al., 2000	11053349	J Appl Physiol (1985)	cross sectional study	20	24.6	2.3		0	22.9	3.1	n.a	us	0.92	0.48									
13	Rakobowchuk et al., 2011	20852882	Eur J Appl Physiol	Interventional study	15	20.6	0.8		47			0	us	0.88	0.31			0.46	0.18			x	x	x
14	Poelkens et al, 2007	17622281	Appl Physiol Nutr Metab	Interventional study	12	71	6.6	63-90	0	29	3.3	0	us	1.09	0.19							x	x	
15	Miyachi et al., 2004	15492301	Circulation	randomized intervention study	28	22	1	20-38	0	22.6	0.7	0	us+apt	0.71	0.08							x	x	
16	Miyachi et al., 2003	12511542	Hypertension	cross-sectional study	32	38.3	1.5	20-60	0			0	us+apt	0.68	0.12							x	x	
17	Luik et al., 1997	9430863	Nephrol Dial Transplant	Case Control	24	65.8	7.4		25	26.1	2.6	n.a	us	0.67	0.38					8.8	4			
18	Liu et al., 2011	20665751	Arthritis Care Res (Hoboken)	Case Control	25	46.2	11.2		88	21.5	1.9	0	us			0.26	0.11							
19	Liu et al., 2011	21174209	Heart Vessels	Interventional study	21	21.3		19-23	0	22.1	2.6	0	us	0.67	0.35									
20	Lin et al., 2021	n.a	Artery Research	Interventional study	34	68.1	2.3		52.9	25.2	1.3	0	us+apt	1.29	0.23							x	x	x
21	Lambert et al., 1997	9369271	Hypertension	Interventional study	32	24.8	4.7			22.2	2.7	1	us	1.02	0.36					24.8	10.15		x	x
22	Kool et al., 1995	8585998	Diabetes Care	Case Control	30	26			36.7			1	us	0.94	0.41					22.4	11.8			
23	Kılıçaslan et al., 2023	37424419	Clin Psychopharmacol Neurosci	Case control	39	35.1	7.3		54	23.1	2.6	1	us	1.28	0.68							x		
24	Gonzales et al., 2014	24268938	J Sci Med Sport	Interventional study	48	22	3	18-31	50	24	3	n.a	us			0.48	0.17	0.62	0.32					
25	Gonzales et al., 2013	23692612	Clin Physiol Funct Imaging	Cross sectional study	21	68	4.4	61–78	57.1	23.4	2.6	n.a	us	1.61	0.8								x	
26	Giltay et al., 1999	10064102	Diabetologia	Cross sectional study	34	25.5	6		50	21	2.4	1	us	0.87	0.26					23.4	9.31		x	

27	Fabry et al., 2011	21494767	European Journal of Clinical Pharmacology	randomized double-blind cross-over	20	25	7		100	21	3	1	apt	1.09	0.56					20.43	9.3			
28	de Groot et al., 2005	16118583	Med Sci Sports Exerc	Case control	8	41	10		50			n.a	us			0.5	0.13					x		
29	De Basso et al., 2015	25882720	Clin Exp Pharmacol Physiol	Case control	66	60.4	12.1	40–80	56.1			0	us	1.17	0.3			0.44	0.16				x	x
30	Credeur et al, 2018	29315921	Exp Physiol	Interventional study	20	46	21			27	4	n.a	us	0.45	0.08							x		
31	Brunt et al., 2016	27270841	J Physiol	Interventional study	10	21.9		18–30	60	22.5	0.6	0	us+apt			0.45	0.08					x		
32	Baykara et al., 2017	28039898	J Ultrasound Med	Interventional study	38	26.5	6.2	18–40	16	25.4	1.3	n.a	us	0.9	0.05					26.06	1.31	x		x
33	Armentano et al., 1995	7607732	Hypertension	Cross sectional case control	16	48	7		0	24	2	1	other	1.69	0.34					12.11	2.51	x		
34	Arcaro et al., 2004	15126920	J Hypertens	Interventional study	12			21–37	0	23.8	0.7	0	us	1.24								x	x	

Apt = applanation tonometry; BMI = body mass index; CC = cross-sectional compliance; CFA = common femoral artery; DC = distensibility coefficient; n.a. = not assessed; PA = popliteal artery; PMID = Pubmed ID; SD = standard deviation; SFA = superficial femoral artery; us = ultrasound

Table S5: Demographic characteristics and extracted study data for studies included in the male subgroup analysis.

No.	Author et al., year	PMID	Journal name	Design	Participants n	Mean age	Age SD	Age range	Female (%)	BMI (mean)	BMI (SD)	Smokers included	CFA CC mean	CFA CC SD
1	Wijnen et al. 1991	2036093	Basic Res Cardiol	Case Control	15	24	1		0			0	1.43	0.12
2	Vermeersch et al., 2008	18551018	Jounal of Hypertension	Cohort study	1064	45.3	1.6	35–56	0			1	0.71	0.02
3	van Vonderen et al., 2009	19131894	J Acquir Immune Defic Syndr	Case Control	52	49.6			0	23.9		1	1.09	0.05

4	Schmidt-Trucksäss et al., 2000	11053349	J Appl Physiol (1985)	cross sectional study	20	24.6 (2.3)			0	22.9	3.1	n.a	0.92	0.48
5	Miyachi et al., 2004	15492301	Circulation	randomized intervention study	28	22	1	20-38	0	22.60	0.70	0	0.71	0.08
6	Miyachi et al., 2003	12511542	Hypertension	cross-sectional study	32	38.3	1.5	20-60	0			0	0.68	0.12
7	Liu et al., 2011	21174209	Heart Vessels	Interventional study	21	21.3	1	19-23	0	22.10	2.60	0	0.67	0.35
8	Poelkens et al, 2007	17622281	Appl Physiol Nutr Metab	Interventional study	12	71	6.6	63-90	0	29.00	3.30	0	1.09	0.19
9	Gonzales et al., 2013	23692612	Clin Physiol Funct Imaging	Cross sectional study	9	72	5	61–78	0	24	2.00	n.a	1.9	1.2
10	Giltay et al., 1999	10064102	Diabetologia	Cross sectional study	17	28	7		0	21.20	2.60	1	0.93	0.27
11	De Basso et al., 2015	25882720	Clin Exp Pharmacol Physiol	Case control	29	61	11	40–78	0			0	1.50	1
12	Armentano et al., 1995	7607732	Hypertension	Cross sectional case controll	16	48	7		0	24.00	2.00	1	1.69	0.34
13	Arcaro et al., 2004	15126920	J Hypertens	Interventional study	12	n.a.		21–37	0	23.80	0.70	0	1.21	1.24

BMI = body mass index; CC = cross-sectional compliance; CFA = common femoral artery; n.a. = not assessed; PMID = Pubmed ID; SD = standard deviation

Table S6: Demographic characteristics and extracted study data for studies included in the female subgroup analysis.

No.	**Author et al., year**	**PMID**	**Journal name**	**Design**	**Participants n**	**Mean age**	**Age SD**	**Age range**	**Female (%)**	**BMI (mean)**	**BMI (SD)**	**Smokers included**	**CFA CC mean**	**CFA CC SD**
1	Willekes et al., 1999	10414888	Ultrasound Med Biol	Controlled quasi-experimental study	14			18-25	100			n.a.	0.8	0.4
2	Willekes et al., 1998	9740382	Ultrasound Med Biol	Controlled quasi-experimental study	24			18-35	100			2	0.67	0.4
3	Vermeersch et al., 2009	18551019	Jounal of Hypertension	Cohort study	1131	44.9	1.6	35–40	100			1	0.81	0.02

4	Gonzales et al., 2013	23692613	Clin Physiol Funct Imaging	Cross sectional study	12	65	4	61–78	100	23	3.00	2	1.4	0.5
5	Giltay et al., 1999	10064102	Diabetologia	Cross sectional study	17	23	5		100	20.80	2.10	1	0.8	0.25
6	Fabry et al., 2011	21494767	European Journal of Clinical Pharmacology	randomized double-blind cross-over	20	25	7		100	21	3.00	1	1.09	0.56
7	De Basso et al., 2015	25882720	Clin Exp Pharmacol Physiol	Case control	37	60	13	40–80	100			0	0.91	0.08

BMI = body mass index; CC = cross-sectional compliance; CFA = common femoral artery; n.a. = not assessed; PMID = Pubmed ID; SD = standard deviation
